# IgA Vasculitis in Japanese Patients Harboring MEFV Mutations: A Case Report and Review of the Literature

**DOI:** 10.7759/cureus.34876

**Published:** 2023-02-11

**Authors:** Tadafumi Yokoyama, Naoto Sakumura, Natsumi Inoue, Yusuke Matsuda, Taizo Wada

**Affiliations:** 1 Pediatrics, Kanazawa University, Ishikawa, JPN

**Keywords:** colchicine, mefv gene, il-18, iga vasculitis, familial mediterranean fever

## Abstract

Immunoglobulin A vasculitis (IgAV) is the most common vasculitis of childhood. However, its etiology remains unknown. In the Mediterranean region, 10% of patients with IgAV harbor homozygous and compound heterozygous mutations in the Mediterranean fever (*MEFV*) gene. Thus, such mutations may be involved in the development of IgAV.

Herein, we present a five-year-old girl presented with IgAV. She experienced prolonged abdominal pain, which was steroid-resistant. When treatment with colchicine was started, her abdominal pain resolved immediately. The serum interleukin (IL)-18 levels of the patient and other patients with IgAV and familial Mediterranean fever (FMF) were evaluated using enzyme-linked immunosorbent assay. The serum IL-18 level of the patient was higher than that of other patients with IgAV and was similar to that of patients with FMF harboring M694I mutation. Moreover, all exons of the *MEFV* gene were analyzed using the Sanger sequencing and the patient presented with E148Q/M694I mutation. Further, a comprehensive search of Japanese patients with IgAV harboring *MEFV* gene mutations in PubMed, Ichushi-Web, and Medical Online was conducted to validate the clinical characteristics of Japanese patients with IgAV harboring *MEFV* gene mutation. In previous studies, only five patients presented with IgAV harboring *MEFV* gene mutation in Japan.

The prevalence of IgAV associated with *MEFV* gene mutation may be low in Japan. However, *MEFV *gene mutations should be suspected if the symptoms of IgAV are prolonged or if patients are refractory to treatment. In such case, IL-18 monitoring and colchicine treatment may be useful for IgAV with *MEFV* gene mutation.

## Introduction

Immunoglobulin A vasculitis (IgAV), traditionally referred to as Henoch-Schönlein purpura or anaphylactoid purpura, is characterized by non-thrombocytopenic palpable purpura, abdominal pain, arthritis, and glomerulonephritis. Moreover, it is the most common vasculitis of childhood [1-6]. The pathogenesis of IgAV remains unknown. However, it is associated with different factors including infections and chemical triggers, galactose-deficient IgA, and low coagulation factor XIII level [1]. To date, a monogenic defect leading to IgAV has not been discovered [1-3].

Familial Mediterranean fever (FMF) is a genetic disease affecting Sephardic Jews, Arabs, Turks, and Armenians and is caused by mutations in the Mediterranean fever (MEFV) gene. Further, it is characterized by recurrent febrile episodes and serous membrane inflammation [1-2, 4, 7]. In communities in which FMF is most common, 85% of genetic mutations were encoded in exons 10 and 2. That is, exon 10 contains four principal mutations (M694V, V726A, M680I, and M694I); exon 2, 1 mutation (E148Q) [1-2, 7]. In studies conducted in Israel and Turkey, patients with IgAV commonly present with MEFV gene mutations [5, 7-9]. Recently, although some reports have not revealed any association [8-9], patients with IgAV harboring MEFV gene mutations present with a more severe clinical course and laboratory findings due to an exaggerated inflammatory response caused by the defective gene [2, 5-7, 10].

There are two types of FMF in Japan [11-12]. First, clinically typical FMF is associated with exon 10 mutations and is characterized by episodes lasting from 12 hours to 3 days and fever accompanied by peritonitis, pleuritis, or monoarthritis of the hip, knee, or ankle [11]. Unlike the mutations observed among individuals in the Middle East, most mutations are M694I homozygote or E148Q/M694I compound heterozygote [11-14]. M694V, V726A, and M680I mutations have not been reported among Japanese individuals [12-14].

Second, atypical or incomplete FMF is not associated with exon 10 mutation among Japanese patients [11]. This condition is characterized by milder symptoms such as temperature of <38℃, attack episodes lasting from 6 h to one week, absence of peritonitis during abdominal pain, localized abdominal signs, and atypical distribution of arthritis [11]. The incidence of typical FMF is <1/250-500 among Japanese patients in highly affected areas [13].

Further, the development of IgAV should be consistent with the geographic distribution of MEFV abnormalities if mutations are involved in IgAV pathogenesis. However, this assumption is not actually true. Importantly, IgAV is a ubiquitous disease with no evident geographical, racial, or ethnic variations in terms of risk [1, 3].

Herein, we report the clinical course of a Japanese patient with IgAV harboring MEFV gene mutation. The epidemiological and clinical features of Japanese patients with IgAV harboring MEFV gene mutation have not been reported in previous studies. Therefore, we also aimed to perform a comprehensive literature analysis of Japanese patients with this condition.

## Case presentation

**Figure 1:**
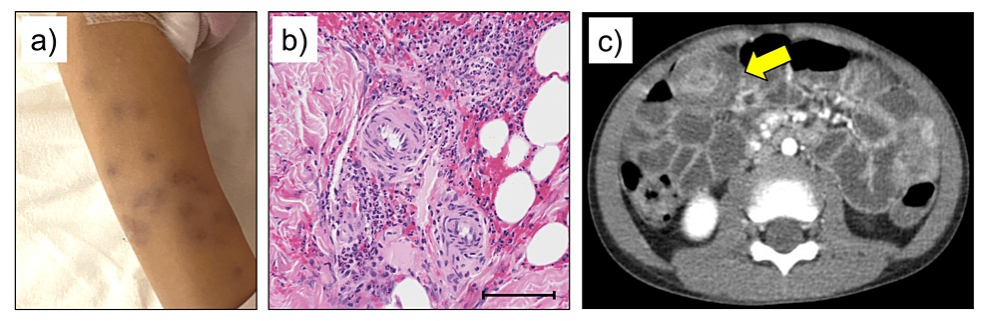
Clinical image of our patient (IgAV associated with MEFV gene mutation). a) Purpura in the lower extremities. b) Skin biopsy of the purpura revealed leukocytoclastic vasculitis (scale bar: 100 μm). However, IgA deposition could not be observed. c) Abdominal CT scan performed at day 23 revealed target signs indicating intussusception (yellow arrow). IgAV, IgA vasculitis; MEFV gene, the familial Mediterranean fever gene

A previously healthy five-year-old girl presented with purpura and edema in both upper and lower extremities (Figure 1a). There was no family history of periodic fever and/or FMF. Her coagulation factor XIII level was 66% (normal: >70%). However, the platelet count was 3.27 × 105/μL, and the other coagulation factor levels were within normal range. The level of serum IgA was 185 mg/dL. Skin biopsy revealed leukocytoclastic vasculitis, but without IgA deposition (Figure 1b). The patient was diagnosed with IgAV by a home physician.

Then, the patient was admitted at our hospital because of abdominal pain, gross hematuria, and urinary retention due to a bladder mass, which was immediately resolved with glucocorticoid treatment, as mentioned by Inoue et al. in a case report [15].

However, purpura, arthritis, and mild abdominal pain persisted. In addition, the patient developed intussusception 23 days after admission (Figure 1c). When the glucocorticoid dose was increased, the intussusception and abdominal pain was relieved without hydrostatic enema reduction. Then, there was recurrence in abdominal pain with weaning glucocorticoid doses.

Abdominal pain gradually could not be controlled by glucocorticoid. And, her abdominal pain changed from continuous to periodic. Her abdominal pain occurred once a day, usually in the morning. The patient was having self-resolving intermittent abdominal pain flare ups. Treatment with diaminodiphenyl sulfone and montelukast was initiated for abdominal pain attack [16-17]. However, it was not effective.

Although the duration and frequency of attacks differed, her abdominal pain was periodic similar to that of FMF. The patient was treated with colchicine (0.3 mg/day; 0.02 mg/kg/day) two months after admission, and abdominal pain episodes disappeared within a few days. Then, therapy with glucocorticoids was tapered and discontinued one month after starting colchicine. Finally, colchicine was also discontinued two months later.

After six months, only purpura was observed again. She did not take any medications. Nevertheless, it improved without abdominal pain within a few days. Since then, she has been continually monitored without any medications for over two years, and had no complications of IgAV such as Henoch-Schönlein purpura nephritis or symptoms of FMF-like periodic fever and abdominal pain.

Evaluation of serum interleukin-18 levels

The serum interleukin (IL)-18 level was assessed using enzyme-linked immunosorbent assay (ELISA) (MBL Human IL-18 ELISA Kit, MBL International Corporation, Woburn, MA) [2]. To compare serum IL-18 levels of our case, we evaluated the serum IL-18 levels of eight healthy children (control), four symptomatic IgAV patients; having purpura and/or abdominal pain, five with typical FMF (before starting colchicine, afebrile phase) harboring E148Q/M694I compound heterozygote mutation (FMF E148Q/M694I), and six with atypical FMF without M694I mutation [FMF M694I (-)], which was confirmed using the Tel-Hashomer and Livneh diagnosis criteria [2, 14, 18].

**Figure 2:**
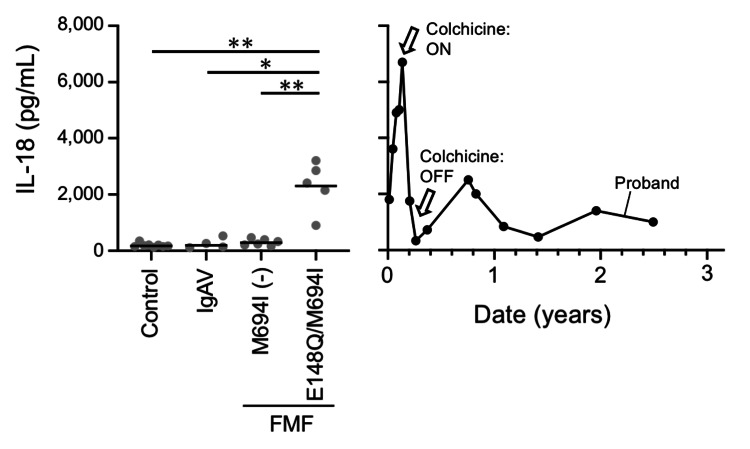
Serum IL-18 levels of our patient, other patients with IgAV, and patients with FMF. The serum IL-18 levels of patients with FMF E148Q/M694I were significantly higher than those of healthy controls, patients with IgAV, and patients with FMF M694I (-) (*: p < 0.05, **: p < 0.005). The serum IL-18 levels of our patient were higher than those of the controls and other patients with IgAV. Moreover, they were similar to those of patients with FMF E148Q/M694I. The serum IL-18 level of our patient decreased to almost normal range after colchicine treatment. In addition, the serum IL-18 level of our patient increased similar to that of patients with FMF E148Q/M694I after colchicine discontinuation. IgAV, IgA vasculitis; FMF, familial Mediterranean fever; IL-18, interleukin-18

Patients with FMF E148/M694I had higher IL-18 levels at the afebrile phase than healthy controls and patients with IgAV and FMF M694I (-) (Figure 2). The IL-18 level gradually increased, which was correlated with abdominal pain intensity. However, when treatment with colchicine was started, the IL-18 level immediately decreased to almost normal range.

MEFV gene mutation analysis

We sequenced all exons of the MEFV gene of the patient and her family. Briefly, after obtaining the consent from the parents and the assents from the patient and brother, 2 mL of blood sample was collected. Genomic DNA was extracted using a DNA extraction kit (QIAamp DNA Blood Mini Kit, Qiagen, Venlo, the Netherlands). Polymerase chain reaction of the genomic DNA was performed using primers specific to each exon of the MEFV gene. Then, the nucleotides of the polymerase chain reaction product were sequenced.

**Figure 3:**
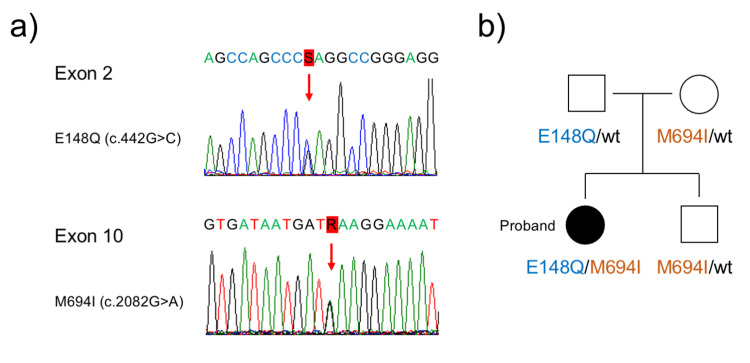
Result of the MEFV gene mutation analysis of our patient and her family members. a) Result of the direct sequencing of the proband’s *MEFV* gene. There were missense mutations at c.442G>C in E148Q in exon 2 and missense mutation at c.20821G>A in M694I in exon 10 of the *MEFV* gene. b) Family tree of the proband. *MEFV* gene, the familial Mediterranean fever gene

The patient presented with E148Q/M694I compound heterozygote mutation (Figure 3a), which is the most common variant among Japanese patients with FMF. However, she did not present with periodic fever. Her father presented with E148Q heterozygote mutation, and her mother and brother with M694I heterozygote mutation in the MEFV gene (Figure 3b). There was no family history of periodic fever and/or FMF.

Assessment of the clinical and biographic characteristics of Japanese patients with IgAV harboring MEFV gene mutation

**Figure 4:**
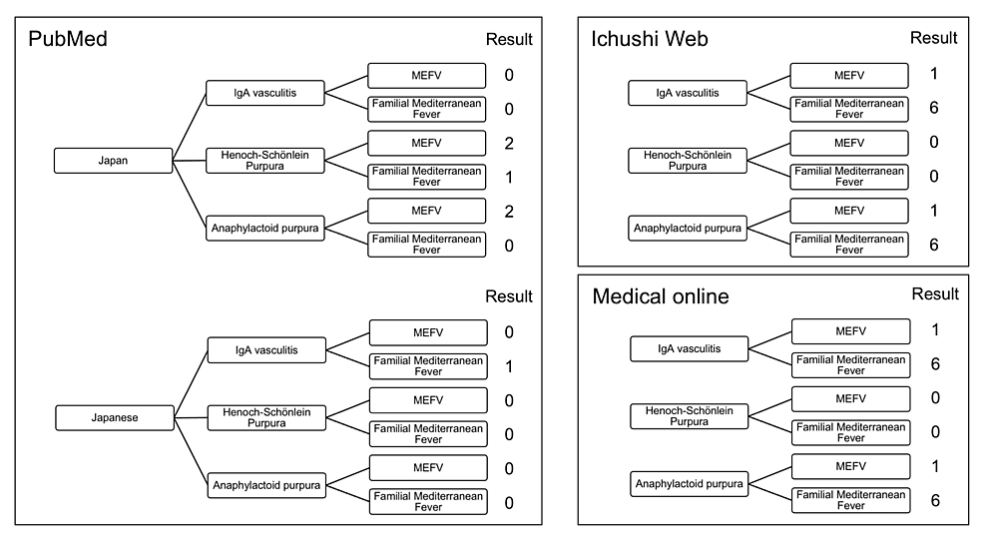
Algorithm and result of the comprehensive literature analysis of Japanese patients with IgAV harboring MEFV gene mutations. In the analysis of studies in PubMed, three keywords are shown in the tree diagram. Two articles were searched using the following keywords: “Japan,” “Henoch-Schönlein purpura,” and “MEFV.” Similarly, one article was searched using the following keywords: “Japan,” “Henoch-Schönlein purpura,” and “Familial Mediterranean Fever.” In total, six studies were detected. In the article analysis using the Japanese article search engine (Ichushi-Web and Medical Online), two keywords were detected in the tree diagrams. Moreover, 14 papers were found in Ichushi-Web and Medical Online.

We investigated the clinical characteristics of patients with IgAV harboring MEFV gene mutation in Japan. We searched for articles in PubMed using the following keywords: "Japanese or Japan” and "IgA vasculitis or Henoch-Schönlein purpura or Anaphylactoid purpura,” and "MEFV or familial Mediterranean fever.” Moreover, medical articles written in Japanese were searched using two search engines, which were as follows: Ichushi-Web (Japan Medical Abstract Society) and Medical online (Meteo Inc.). The search method was similar to that used in PubMed. However, the first keyword (“Japanese or Japan”) was omitted. Figure 4 shows the search algorithm and results.

Six documents (including duplicates) were found in PubMed. By contrast, 28 studies (including duplicates) were found in the Japanese search engine. In these studies, five patients presented with IgAV harboring MEFV gene mutation. Four documents were conference abstracts and one case report [19]. Table 1 depicts information about the other five patients with IgAV harboring MEFV gene mutation and our patient.

**Table 1:** Reported cases about Japanese IgA vasculitis with MEFV mutation. DDS, diaminodiphenyl sulfone; In, ineffective; Partial, partially effective; MTX, methotrexate; IgAV, immunogloblin A vasculitis; FMF, familial Mediterranean fever; MEFV, Mediterranean fever, N.D., not described

Case	Age	Sex	Course	Duration from first symptom to diagnosis of IgAV with MEFV gene mutation	Symptoms	Colchicine	Other treatment	MEFV gene mutation	Year	Authors	Reference
1	9	Female	Periodic fever → Vasculitis	Over 4 years	Periodic fever and vomiting since infant. At 5 y, tonsillectomy. At 9 y, refractory purpura.	Effective	Tonsillectomy (In)	P369S/R408Q	2020	Kubota et al.	Conference abstract written in Japanese
2	52	Female	Periodic fever → Vasculitis	Over 40 years	Periodic fever since childhood. At 34 y, recurrent arthritis and diagnosed as FMF. Recurrent purpura for 6 years.	Ongoing	MTX	N.D.	2014	Yamamoto et al.	Conference abstract written in Japanese
3	49	Male	Vasculitis → Prolonged symptoms	8 years	At 41 y, purpura, arthritis, abdominal pain, vasculitis, and purpura nephritis. At 49 y, shortening of period of fever and abdominal pain.	Effective	Steroid (In), Immunosuppressant (In)	E148Q/M694I	2014	Sato et al.	Conference abstract written in Japanese
4	3	Male	Vasculitis → Prolonged symptoms	6 months	Purpura, abdominal pain, arthritis, and decreased platelet. Refractory fever for 6 months and hepatosplenomegaly.	Ongoing	Steroid (In), Antibiotics (In)	G304R/wt	2015	Kinoshita et al.	Conference abstract written in Japanese
5	51	Male	Periodic fever → Vasculitis	Over 40 years	Periodic fever since childhood. At 46 y, diagnosed as IgAV based on skin biopsy. Recurrent purpura for 6 years.	Effective	Steroid (Partial), Immunosuppressant (In)	E148Q/M694I	2021	Sasajima et al.	[19]
6	5	Female	Vasculitis → Prolonged symptoms	1.5 months	Purpura and fever, bladder mass, intussusception. Refractory abdominal pain for 2 months.	Effective	Steroid (In), DDS (In), Montelukast (In)	E148Q/M694I	Our case	Our case	Our case

Three patients (cases 1, 2, and 5) diagnosed with periodic fever developed IgAV. Interestingly, cases 1 and 5 were not diagnosed to MEFV gene mutation although they had periodic fever which looks FMF symptom in their childhood. And, MEFV gene analysis was performed after their suffering IgAV. The other two patients (cases 3 and 4) were diagnosed with IgAV before the onset of periodic fever. In such cases, the patients were treated with colchicine six months to two years after the diagnosis of IgAV. Meanwhile, our patient was treated with colchicine 1.5 months after diagnosis, which was the fastest. The five patients received treatments such as tonsillectomy and use of methotrexate, steroids, and immunosuppressant. They were refractory to these treatments. However, they responded to colchicine (0.5 mg-1.0 mg/day).

## Discussion

To the best of our knowledge, this literature review first described IgAV associated with *MEFV *gene mutation in Japanese patients. In our case, IgAV was clinically diagnosed based on the presence of palpable purpura, leukocytoclastic vasculitis, which was confirmed via skin biopsy, and intussusception. In the early stages of the disease, abdominal pain was manageable with steroids. However, it gradually became steroid-dependent and, eventually, steroid-resistant. Various treatments were provided. However, the patient was refractory. Then, colchicine was administered because abdominal pain was periodic and very intense. Steroid was ineffective, and colchicine was highly effective. Thus, we hypothesized her pathological condition was similar to FMF.

FMF is a genetic abnormality associated with inflammasome, and patients with this condition present with high serum IL-1β and IL-18 levels [11,14,20]. In particular, FMF with M694I mutation is associated with high IL-18 levels even in the intermittent afebrile phase [11,20]. In the current case, the serum IL-18 levels of our patient were higher than those of other patients with IgAV, and they decreased with the administration of colchicine. Interestingly, after the discontinuation of colchicine, the IL-18 levels increased again. However, its level was equivalent to that of patients with FMF M694I (+). During this period, the patient had no symptoms of FMF and no elevation of blood inflammatory markers like C-reactive protein (CRP) and serum amyloid A (SAA).

To the best of our knowledge, this literature review first described IgAV associated with MEFV gene mutation in Japanese patients. In our case, IgAV was clinically diagnosed based on the presence of palpable purpura, leukocytoclastic vasculitis, which was confirmed via skin biopsy, and intussusception. In the early stages of the disease, abdominal pain was manageable with steroids. However, it gradually became steroid-dependent and, eventually, steroid-resistant. Various treatments were provided. However, the patient was refractory. Then, colchicine was administered because abdominal pain was periodic and very intense. Steroid was ineffective, and colchicine was highly effective. Thus, we hypothesized her pathological condition was similar to FMF.

Familial Mediterranean fever is a genetic abnormality associated with inflammasome, and patients with this condition present with high serum IL-1β and IL-18 levels [11, 14, 20]. In particular, FMF with M694I mutation is associated with high IL-18 levels even in the intermittent afebrile phase [11, 20]. In the current case, the serum IL-18 levels of our patient were higher than those of other patients with IgAV, and they decreased with the administration of colchicine. Interestingly, after the discontinuation of colchicine, the IL-18 levels increased again. However, its level was equivalent to that of patients with FMF M694I (+). During this period, the patient had no symptoms of FMF and no elevation of blood inflammatory markers like C-reactive protein (CRP) and serum amyloid A (SAA).

Based on the clinical characteristics and serum IL-18 levels, we considered the possibility of MEFV gene mutations in this patient. In fact, she presented with E148Q/M694I compound heterozygote mutation.

Hence, MEFV gene mutation was a symptom modifier in this patient. However, in a strict sense, whether this patient presented with clinically typical FMF remains unclear. This is because she did not have recurrent febrile episodes. FMF may develop in adulthood, and the IL-18 concentration in this patient is still high even though asymptomatic [11, 20]. Therefore, she should be monitored for periodic febrile episodes in the future.

No study has investigated the epidemiological or clinical features of IgAV associated with MEFV gene mutation in Japanese patients. IgAV is predominantly a childhood disease, with an incidence of 3-26.7 cases per 100,000 children [3, 5, 7]. IgAV is a ubiquitous disease with no evident geographical, racial, or ethnic variations in terms of risk [3].

By contrast, there are significant differences in the incidence rate of MEFV gene mutation. In Turkey, Israel, and Armenia, field surveys revealed that the prevalence of FMF is approximately 1 in 500-1000 children (0.1%-0.2%) [5, 7, 9]. The number of patients with FMF in Japan is about 500 (1 in 1-5×105: 0.0002%-0.001%) and <1/250-500 in highly affected areas. Moreover, 25% of individuals in the general population in Japan present with E148Q heterozygote mutation. Thus, E148Q is considered a single nucleotide polymorphism, and FMF can develop when E148Q and M694I cause compound heterozygote mutation [20].

If the MEFV gene is not involved in IgAV pathogenesis, the prevalence of IgAV among patients with MEFV gene mutation will be similar to that among patients without MEFV gene mutation in the general population. However, in Turkey, 10% of patients with IgAV presented with homozygote/compound heterozygote mutations in the MEFV gene. The rate is significantly higher than that of MEFV gene mutations in the general population [5, 8]. The clinical features in IgAV with MEFV gene mutations are 1) younger, 2) severer edema, arthritis and abdominal pain, 3) refractory against conventional IgAV treatment, 4) elevated CRP and SAA, and 5) lack of IgA deposits in skin biopsy [5, 8].

In Japan, several patients with IgAV could present with MEFV gene mutations. However, previous studies have not reported comprehensive results, and there are only five patients found in the literature review. In addition, there is no report showing that IgAV is likely to occur in Japanese patients with FMF. Whether MEFV gene mutation is associated with the pathophysiology of IgAV among Japanese, similar to those in the Mediterranean region, remains unclear. Nevertheless, a cohort study must be conducted to validate the clinical and epidemiological characteristics of IgAV correlated with MEFV gene mutation among Japanese.

This article was previously posted to the Research Square preprint server on April 14, 2022.

## Conclusions

The prevalence of IgAV associated with MEFV gene mutations in Japan may be low. However, MEFV gene mutations can be masked if symptoms are prolonged or if patients are refractory to treatment. In such cases, IL-18 monitoring may be useful, and colchicine can be a treatment option for refractory IgAV.
